# Mercury Determination in Fish Samples by Chronopotentiometric Stripping Analysis Using Gold Electrodes Prepared from Recordable CDs

**DOI:** 10.3390/s8117157

**Published:** 2008-11-12

**Authors:** Maria-Cristina Radulescu, Andrei Florin Danet

**Affiliations:** University of Bucharest, Faculty of Chemistry, Sos. Panduri 90-92, 050657, Bucharest, Romania; E-Mail: cristinariz2002@yahoo.com

**Keywords:** Mercury analysis, gold electrodes, chronopotentiometric stripping analysis, fish digestion, fish analysis

## Abstract

A simple method for manufacturing gold working electrodes for chronopotentiometric stripping measurements from recordable CD-R's is described. These gold electrodes are much cheaper than commercially available ones. The electrochemical behavior of such an electrode and the working parameters for mercury determination by chronopotentiometric stripping analysis were studied. Detection limit was 0.30 μg Hg/L and determination limit was 1.0 μg Hg/L for a deposition time of 600 s. Using the developed working electrodes it was possible to determine the total mercury in fish samples. A method for fish sample digestion was developed by using a mixture of fuming nitric acid and both concentrated sulfuric and hydrochloric acids. The recovery degree for a known amount of mercury introduced in the sample before digestion was 95.3% (n=4).

## 1. Introduction

Platinum electrodes have been among the mostly used solid metallic electrodes for electroanalytical determinations. This material is preferred in principal due to its “inertia” in the presence of numerous chemical species. Gold electrodes have become more used in practice because of two important applications: stripping analysis of several metallic ions (such as: Cu(II) [1, 2], Pb(II) [2, 3], As(V) [4, 5]) and studies implying their surface modifications based on self-assembling of some specific compounds [1, 2, 6, 7].

In the stripping analysis of mercury, gold electrodes produce well-defined peaks so that low detection limits can be achieved [8-11]. Wang *et al.* [12] studied screen-printed carbon strip electrodes on which a thin gold film was deposited in order to determine mercury by potentiometric stripping by using a gold film working electrode. Mercury determination by means of these very cheap, disposable electrodes is done under conditions comparable with those for conventional gold electrodes.

Gil and Ostapczuk [13] determined mercury as well as other heavy metals (Se(IV), Cu(II) and Pb(II)) by potentiometric stripping analysis. Anodic stripping has been done by an oxidation constant current that follows the reductive deposition of the elements in atomic form on the working electrode.

Golimowski *et al.* [14] employed gold disk electrodes for determining mercury in residual waters by a voltammetric method. Daniele *et al.* [15] made a system for remote monitoring of mercury by potentiometric stripping and square wave stripping voltammetry by means of gold wire microelectrode. This analytical method was used for mercury determination in the Venetian Laguna.

Commercially available gold electrodes are quite expensive and require several steps of mechanical and/or electrochemical cleaning prior to analysis. This is especially valid in the case of determinations of mercury which can easily penetrate the gold layer due the formation of amalgam.

The gold electrodes for analytical applications can be manufactured by electrolysis deposition of gold on a glassy carbon electrode [16] or by other deposition procedures on different supports [10-12]. Deposition methods are however laborious and difficult enough so that the obtained electrodes can be used for only a reduced number of experimental runs.

This was the reason why good quality gold electrodes obtained by a simple method at a reduced price have become interesting for mercury determination. A source for gold electrodes was represented by recordable compact disks (CD-R's), which have a gold film as a reflecting layer. It is worth noticing the fact that several studies were reported in literature regarding the gold electrode manufacturing starting from CD-R's [17-20] and their use in mercury determinations by chronopotentiometric stripping analysis. This type of electrodes were used also for amperometric determination of ascorbic acid [21] and dipyrone [22] in pharmaceutical formulations, for on-line amperometric determination of Ce(IV) [23] during polymerization reactions, for amperometric detection [24] in capillary electrophoresis for determination of Cu(II) and Pb(II) [25] in lubricating oils by using square-wave anodic stripping voltammetry, for determination of Cu(II) [26] in sugar cane spirits and tap waters, by chronopotentiometric stripping analysis, for developing small flow cells for voltammetry and flow injection analysis [20-22, 27, 28].

In this paper was presented a study on gold electrode utilizing CD-R's from Kodak [29] and the use of such electrodes for mercury determinations by stripping chronopotentiometry. A method for digestion of fish sample by using fumans nitric acid 100% was also optimized. The developed stripping chronopotentiometric method was applied for determination of mercury in fish samples. At present mercury is determined in fish samples especially by cold vapor atomic absorption spectrometry (CVAAS) [30, 31]. Chronopotentiometric stripping analysis comparatively with CVAAS has an important advantage in the determination of mercury namely: the necessary equipment is much cheaper and smaller and the cost of an analysis is low.

## 2. Experimental

### 2.1. Materials and Reagents

Nitric acid 65%, fuming nitric acid 100%, sulfuric acid 98%, hydrochloric acid 37% and hydrogen peroxide 30% were purchased from Merck (Darmstadt, Germany). Crystallized copper sulfate was obtained from Reactivul (Bucharest, Romania). Standard mercury solution of 1000 mg/L for atomic absorption spectrometry was obtained from Hg(NO_3_)_2_ in HNO_3_ 2M (Fluka). An intermediary standard of a 10 mg Hg/L concentration was prepared from the above solution by dilution with 0.05 M HCl solution. This solution was prepared on a weekly basis. A standard solution of 0.1 mg Hg/L was prepared daily by diluting with HCl 0.05 M the intermediary standard of 10 mg Hg/L concentration. Electroconductive paste and fast hardening epoxy resin, both from Bison, Fulda-Germany, were used for preparing of the gold electrodes. Solutions of 0.2 M H_2_SO_4_ and 0.05 M HCl were prepared by diluting the corresponding volumes of concentrated acids with bidistilled water. A Cu(II) solution of 1000 mg Cu(II)/L was prepared by dissolving the corresponding amount of CuSO_4_·5 H_2_O in bidistilled water. The 10 mg Cu(II)/L solution was then prepared by diluting the corresponding volume of 1000 mg Cu(II)/L solution with 0.05 M HCl. The working solutions of Cu(II) were prepared by diluting the latter with 0.05 M HCl solution. All vessels were kept in water with detergent for approximately 16 hours and then washed with tap water. They were subsequently kept for another 8 hours in 6 M nitric acid and washed again with tap, distilled and bidistilled water.

### 2.2. Apparatus

All experiments were carried out by means of a PalmSens potentiostat from Palm Instruments BV-The Netherland coupled to a Pentium IV PC for data acquisition and processing. An electrochemical cell (total volume of 80 mL) with three electrodes was employed: gold electrode made from a Kodak recordable CD as working electrode, a saturated calomel electrode as reference electrode and a platinum spiral foil as auxiliary electrode.

### 2.3. Working electrode manufacturing from recordable CD

**Figure 1.**
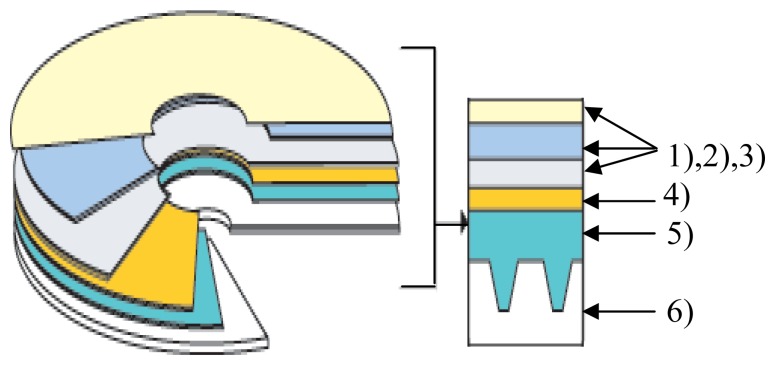
Structure of a CD-R: 1), 2) and 3) protective polymer layers; 4) reflective gold layer; 5) phtalocyanine layer; 6) polycarbonate support. Proportions in the representation were not maintained.

Kodak Gold Preservation (code 1721745) CD-R's were used for our studies (not previously used for the preparation of gold working electrode). The structure of such a CD-R is shown in Figure 1.

Such a CD-R is constituted [29] of a *polycarbonate support* with adequate optical properties on which several layers were deposited:

- A phtalocyanine layer. The phtalocyanine deposited according to a patented procedure is extremely stable within a large domain of environmental conditions, is less sensitive to UV light and solar radiations and reacts faster than other colorants at CD writing.
- A reflective gold layer with thicknesses between 50-100 nanometers and an area of approximately 100 cm^2^ for a CD. The gold used was 100% pure.
- Three protective polymeric layers, scratch proof and of high durability, which offer better protection for handling the disks.

The polycarbonate support is provided with a spiral like groove of very small dimensions spread on the whole writable CD surface. This groove is a guide for the laser radiation on both CD writing and reading. The laser radiation warms up the phtlocyanine layer deposited on the polycarbonate support. When heated over a certain temperature, the phtalocyanine layer is melted and becomes opaque, it is “burnt” (reflects radiation much less than “non-burnt” zones) and a series of “holes” or “pits” are formed. These “pits” are extremely stable physically and are ideal for stocking the data for long periods of time. The information is thus stocked on the CD as burnt and non-burnt zones.

In order to manufacturing gold electrode from a recordable CD, the three protective polymer layers were firstly removed. The CD was placed in an adequate Petri vessel, treated with concentrated nitric acid for a few minutes and then flushed with water to remove the acid. Concentrated nitric acid attacks the protective layers but it has no action on either polycarbonate or gold layers.

**Figure 2.**
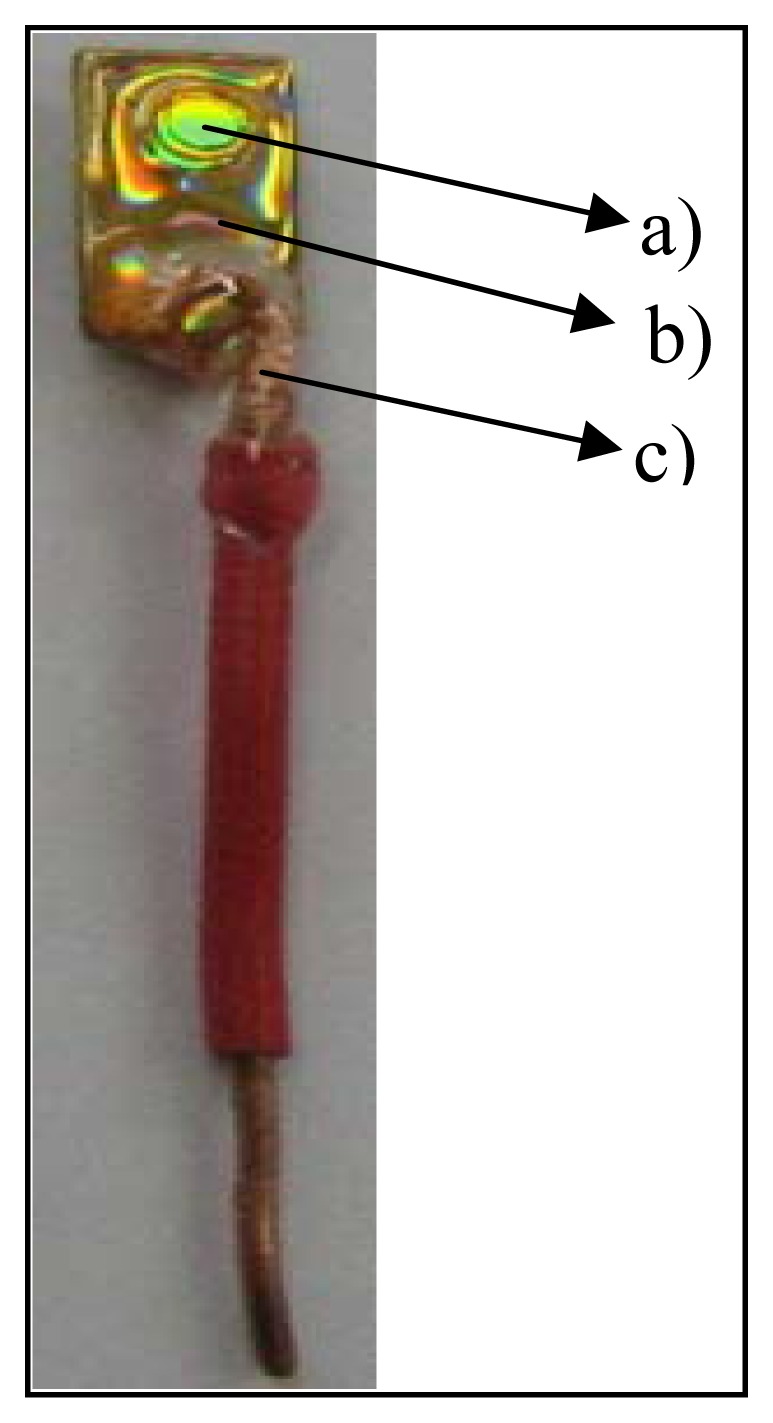
Gold electrode made in the laboratory: a) uncovered gold surface; electroactive surface; b) epoxy resin (covers the whole electrode with the exception of its active surface; c) insulated copper wire of approximately 0.3 cm in diameter.

After removing the protective layers, the CD was cut into pieces of 0.8 cm width and 1.5 cm length. The next step was to delimitate the area which constitutes the gold electrode. In order to do this, a plastic tube of a well-defined diameter was secured on the gold surface and an epoxy resin circle was drawn around the tube. The electric contact of the electrode surface was made by positioning a copper wire at the other end of the electrode and application of electroconductive paste. The whole electrode was then covered with epoxy resin with the exception of the working surface. A gold electrode was thus obtained of a 0.3 cm diameter and of about 0.070 cm^2^ area. Such an electrode is depicted in Figure 2.

The developed electrode was secured on an adequate support provided with an electrical cable (contact pin MP3-type from Metrohm) which connects the electrode to the potentiostat.

### 2.4. Working procedure

An electrochemical cleaning of the working electrode has been done first: 0.2 M H_2_SO_4_ solution (10 mL) was placed in the electrochemical cell and the potential was scanned five times in between -0.25 ÷ +1.5 V. The potential scanning rate was 0.02 V/s. Usually, the last three-recorded voltammograms are practically identical (they overlap each other) and that indicates a proper electrochemical cleaning of the electrode in the first two scannings. Cleaning was repeated if the last two voltammograms do not overlap. After that, electrodes were thoroughly cleansed with bidistilled water.

In order to perform a mercury determination analyte sample (10 mL) prepared in 0.05 M HCl was introduced in the electrochemical cell, stirring was started and working electrode potential was set at 0.3 V for 180 s. After that, stirring was stopped and 15 s were left for equilibration. The potential was then scanned in between 0.3 V and +0.7 V under the conditions when a constant current of +0.75 μA was applied to the working electrode. The signal for a comparison sample (0.05 M HCl solution) was registered at first and it was subsequently subtracted from the signal values registered for all analysed samples. The measured area of the registered peak was expressed in V^2^/s. After each experiment the electrode was cleaned electrochemically by introducing it in a solution of 0.2 M H_2_SO_4_ and by applying a potential of 1.5 V for 40 s.

## 3. Results and Discussion

### 3.1. Electrochemical behavior and surface properties of the manufactured electrode

**Figure 3.**
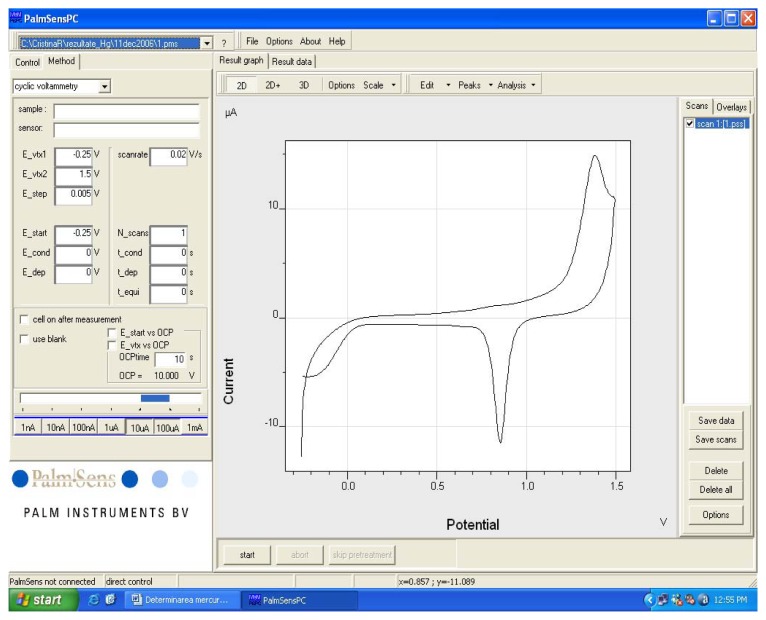
Cyclic voltammogram registered with a gold electrode prepared from a CD in a 0.2 M H_2_SO_4_ solution. Potential scanning was in the domain -0.25 V to +1.5 V and scanning rate was 0.02 V/s.

A cyclic voltammogram registered in 0.2 M H_2_SO_4_ for an electrode made from a CD-R is presented in Figure 3. The cyclic voltammogram was registered according to the working procedure described above. Potential scanning was in the domain -0.25 V to +1.5 V at a scanning rate of 0.02 V/s. One can notice an anodic peak at around 1.37 V corresponding to gold oxidation and a cathodic peak at approximately 0.85 V corresponding to gold oxide reduction. The voltammogram in Figure 3 is similar with the one recorded in the same conditions as reported in literature [28] when a commercially available disk gold electrode (of 3.15 mm^2^ area) was employed. The conclusion was that gold electrodes made from a CD-R behave electrochemically very similar to the commercially available one.

### 3.2. Electrochemical cleaning

An electrochemical cleaning of the working electrode was done before each determination. The literature data [17] recommend an electrode cleaning in a 0.05 M HCl solution at a potential of 0.85 V for 10 s. When we employed this cleaning method, a fast deterioration of the gold layer on the electrode surface was noticed after approximately 20 experimental runs. For this reason, the cleaning of the gold electrode was carried out in a 0.2 M H_2_SO_4_ solution for 40 s at a potential of 1.5 V. Thus, an efficient cleaning of the electrode surface was achieved without any degradation and therefore that electrode can be used for at least 40 runs.

### 3.3. Influence of the potential applied to the working electrode for mercury deposition from solution

The literature data indicate that mercury can be reduced on gold within a wide potential range that is between -0.3 V and 0.3 V [17-19]. This domain can be either wider or narrower as a function of the electrolyte nature and composition of the solution where the reduction process occurs. Some other metals, such as Cu(II), Pb(II), Se(IV)) or other compounds, present in the analysed sample, can be also reduced on the electrode surface along with the mercury. The more positive the potential applied to the working electrode, the less species are reduced on the electrode surface. We have chosen a value of 0.3 V for the deposition potential of mercury in order to avoid co-deposition of the other species from solution and to enhance the determination selectivity as well.

### 3.4. Influence of the supporting electrolyte solution on the analytical signal

For mercury determination by stripping chronopotentiometry when using a gold film electrode, the supporting electrolyte has to contain some species able to complex Hg(II) because the interaction between mercury and gold is very strong and it is difficult to redissolve it. A medium of 0.05 M HCl was chosen.

### 3.5. Influence of the dissolution current on the analytical signal

The best results were obtained when the potential variation measurements for working electrode were carried out in the domain 0.3 V and 0.7 V, while the imposed constant current flew through it. At some more positive potential values, electrochemical dissolution of the gold layer from the electrode surface takes place.

The fundamental equation for chronopotentiometry is the Sand Equation (1):

(1) $$\tau^{1/2}=\frac{\pi^{1/2}{\text{nFCAD}}^{1/2}}{2i}$$

where *τ* is transition time in seconds, *i* is current in μA, *A* is electrode area in cm^2^, *C* is concentration of the electroactive species in mol/cm^3^, *D* is diffusion coefficient of the electroactive species in cm^2^/s and *F* is Faraday number.

The value of the constant current imposed for redissolution of the mercury deposited on the gold electrode surface is an important parameter in the case of chronopotentiometric analytical method. According to Sand's equation [32], when the intensity of the imposed redissolution current was smaller, transition time was longer and therefore sensitivity of the measurement was increased. Yet, a decrease in the redissolution current was accompanied by an important influence of the background noise on the analytical signal. A compromise value has then to be chosen for the intensity of the imposed redissolution current.

**Figure 4.**
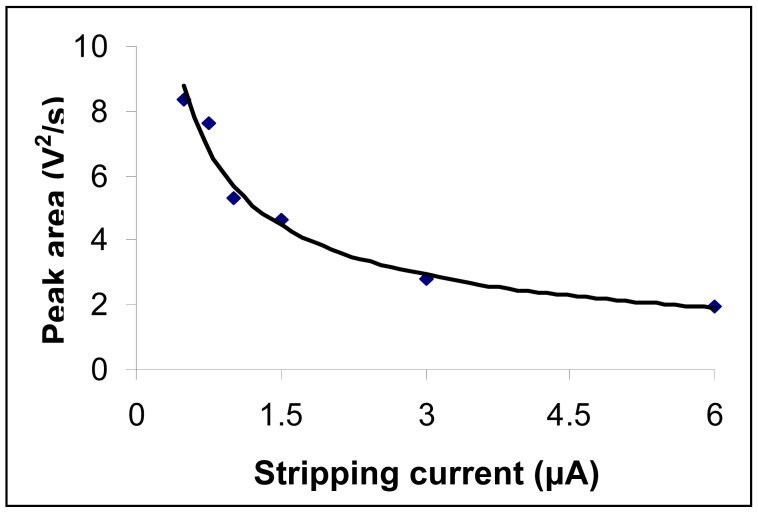
Influence of redissolution current applied to the working electrode on the mercury analytical signal. Mercury concentration was 50 μg/L. Experimental conditions were as described at the working procedure.

Peak areas registered as a function of intensity of the imposed redissolution current when a 50 μg Hg/L solution was employed are depicted in Figure 4. Experimental conditions were the same as those described at the working procedure. Three determinations were done for each current value and a mean value is represented. In Figure 4 it can be seen that an increase of the redissolution current brings about a decrease of the analytical signal. A value of 0.75 μA for the redissolution current was considered as optimal such as to ensure an adequate sensitivity for mercury determination with only a weak influence from the background noise on the registered signal.

### 3.6. Influence of the electrolysis deposition time of mercury on the registered analytical signal

**Figure 5.**
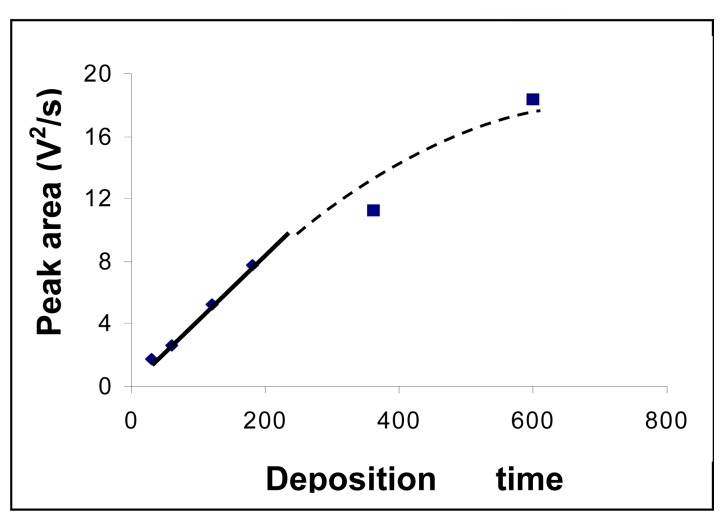
Influence of the electrolysis deposition time of mercury on the gold electrode surface on the registered analytical signal. Concentration of mercury solution was 50 μg/L. Experimental conditions were as described at the working procedure.

The influence of the mercury deposition time onto the gold electrode surface was studied within 30 – 600 s range. A linear dependence between deposition time and registered signal was found in the range 30 – 200 s. The obtained data are presented in Figure 5.

Although determination sensitivity increased with mercury deposition time, a value of 180 s for the deposition time was used in order to do not increase too much the durations of the analysis.

### 3.7. Drawing the calibration graph for mercury determination

**Figure 6.**
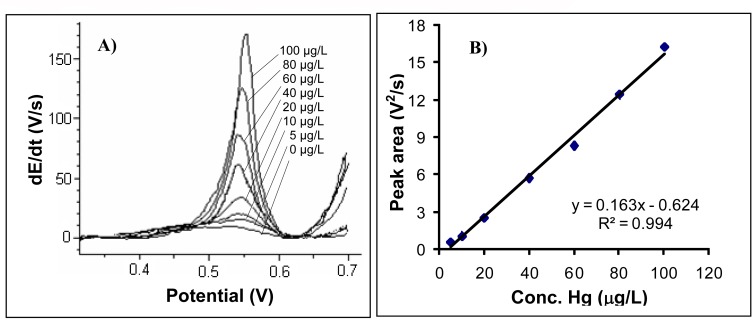
**A**) The chronopotentiograms registered for analysed mercury concentration of: 0; 5; 10; 20; 40; 60; 80; 100 μg/L. **B**) The calibration graph for mercury determination. Experimental conditions were as described at the working procedure.

The chronopotentiograms registered for each analysed mercury concentration are shown in Figure 6A. A calibration line drawn within 5 – 100 μg Hg/L range is given in Figure 6B. The gold electrode surface was cleaned after each run in a 0.2 M H_2_SO_4_ solution for 40 s at a potential of 1.5 V. The straight-line equation was: y = 0.163x – 0.624 and R^2^ = 0.994. Standard relative error calculated for a concentration of 20 μg/L was 1.92% (n=6).

At a deposition time of 600 s a linear dependence between the registered signal and mercury concentration was found within 1 – 10 μg/L concentration range. The experiments were performed according to the described working procedure.

At a deposition time at 600 s, the detection limit was 0.30 μg/L (three times the standard deviation corresponding to a blank sample) and the determination limit (ten times standard deviation for a blank sample) was 1.0 μg/L.

It has been stated that, when the experimental conditions described at the working procedure were employed, a gold electrode made from a CD-R can be used without significant errors for at least 40 experimental runs (although we established that this number could be considerably higher).

### 3.8. Influence of the working electrode area

When a smaller gold electrode (diameter ∼0.2 cm and area ∼0.031 cm^2^) was used, mercury was determined within a 10 – 100 μg Hg/L domain and the obtained signals were proportionally smaller than those for an electrode with a larger surface. The working procedure was as above and calibration straight line equation was: y = 30.494x+0.478 and R^2^ = 0.977. We consider that working with an electrode with a smaller area implies a smaller signal and that implies in more significance of the noise.

### 3.9. Interferences of other ions in mercury determination

**Figure 7.**
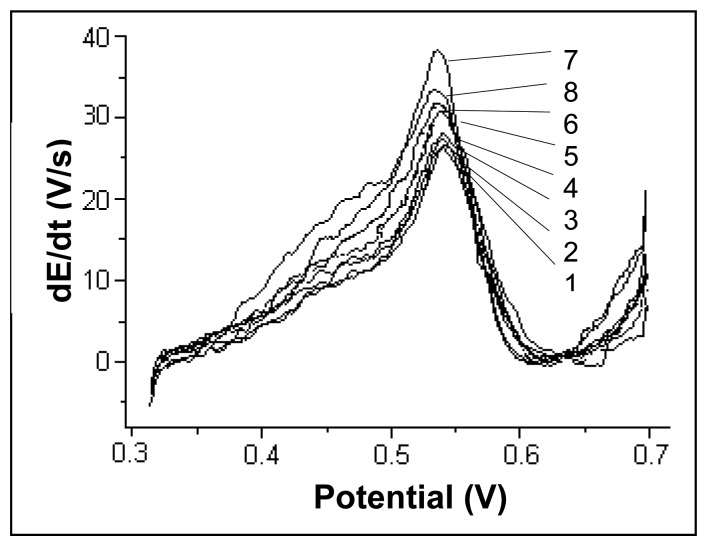
Chronopotentiograms registered for a mercury concentration of 20 μg/L to which different amounts of Cu(II) were added. The ratios C_Cu(II)_/C_Hg(II)_ were of : 0 (1); 1(2); 2 (3); 5 (4); 10 (5); 20 (6); 25 (7); 50 (8). Experimental conditions were as described at the working procedure.

Literature data [17] suggest that Cu(II) ions, with a deposition potential close to the one of Hg(II), can interfere in mercury determination. In Figure 7, the chronopotentiograms registered for a mercury concentration of 20 μg/L are presented when different amounts of Cu(II) were added to obtain final Cu(II) concentrations of 0, 20, 40, 100, 200, 400, 500 and 1000 μg/L.

The mercury signal begins to be slightly affected when Cu(II) concentration becomes higher than 100 μg/L for a C_Cu(II)_/C_Hg(II)_ ratio of 5:1. As Cu(II) ion concentration increases, one can notice the apparition of a shoulder at a potential value of approximately 0.45 V which can be attributed to Cu(II) ions, as well as a signal increase at a potential value of about 0.54 V where the peak maximum for mercury is located. One may consider that under given working procedure conditions, Cu(II) ions do not interfere in mercury determination (errors less than 5%) up to a C_Cu(II)_/C_Hg(II)_ ratio of 5:1.

The following ions do not interfere in the mercury determination till a C_Me(n+)_/C_Hg(2+)_ of 25: Zn(II), Pb(II), Fe(II), Mn(II) (errors less than 5%). The study of the interferences of other ions was carried out using standard solutions and not solutions simulating the ones coming from the acid digestion of fish sample.

### 3.10. Analysis of fish samples

Fish samples (cod) were purchased in a local supermarket and kept in a freezer at -20°C. Prior to digestion, the samples were kept for one hour at room temperature. The muscle of the three fish was then removed from the spinal cord for analysis. The obtained fish fillet was cut into very small pieces with a stainless steel knife working on clean polyethylene surface. Water excess was removed with filter paper. Approximately 50 g from the fish fillet was homogenized in a mortar. Finally, from thus obtained fish paste were extracted the samples which followed the digestion procedure.

The samples were digested adapting to a receipt from literature [33] as follows: fish (cod, 2 g) was weighed in a 250 mL Erlenmayer flask, and then 100% nitric acid (fuming, 5 mL), 98% sulfuric acid (2.5 mL) and 37% HCl (1 mL) were added. The flask was covered with a watch glass and when the reaction stopped, after approximately 20 min, placed on a water bath at 100°C. After one hour the flask was left to cool down. The residual solution (with the volume of about 3.5 mL) was transferred with bidistilled water to a 50 mL volumetric flask. No precipitate appeared on diluting the digested samples with bidistilled water. Samples of 3 mL were extracted from this solution, diluted to 10 mL with 0.05 M HCl and then analysed. If the sample digestion on the water bath last only 40 min (as specified in the cited paper [33]) a precipitate may appear on diluting the digested samples with bidistilled water.

It is known that in oceanic fish mercury [31] exist especially as methyl mercury but during digestion it is converted totally to mercury(II) owing to the strong oxidant action of fumans nitric acid.

As the acids used for sample digestion (although of the best quality) still contain a certain mercury amount and sample matrix was complex enough, the standard addition method was employed for mercury determination.

A comparison sample (containing only the reagents and bidistilled water), a fish sample and another fish sample to which 0.6 μg mercury (as standard solution) were added before digestion were mineralized (by using the above mentioned procedure). Four replicates from each sample were analysed and the mean value computed. The samples were also analysed by cold vapor atomic absorption spectroscopy (CVAAS) according to a method described in literature [34].

A single peak corresponding to mercury was obtained in fish sample analysis by potentiometric stripping analysis. This can be explained by the absence of the interfering ions in the analysed sample. However, the obtained signals (their surfaces) corresponding to the digested fish samples are bigger by approximately 20% than those for synthetic solutions of the same concentrations, but they are similarly well defined. These can be explained in terms of matrix effects.

#### Standard addition method

The fish sample was digested by using the above mentioned procedure. Then to five digested samples (3 mL) placed in five 10 mL volumetric flasks were added standard mercury solution (0, 1, 2 and 4 mL, respectively, with the concentration of 0.1 mg/L) and the volume of the solution was brought to 10 mL with 0.05 M HCl. The concentration of added mercury was of 0, 10, 20 and respectively, 40 μg/L in the final solution. All the samples were analysed by using the described working procedure. By representing the peak area *vs.* added mercury concentration a straight line which intersects the ordinate was obtained. A concentration of 0.239 μg Hg/g in fish sample was determined by using standard addition method.

The results obtained using the method here presented were compared with those obtained with cold vapor atomic absorption spectrometry. These results were in a good agreement and are presented comparatively in Table 1. The standard addition method was also applied to calculate the recovery degree for a known amount of mercury introduced in the fish sample before digestion. A value of 97.6% recovery was founded.

## 4. Conclusions

A method for manufacturing gold working electrodes from recordable CD-R's for chronopotentiometric measurements was presented. Electrochemical behavior of such an electrode and mercury determination by chronopotentiometric stripping analysis using this new type of gold electrode is discussed. The following working parameters for mercury determination by chronopotentiometric stripping analysis were studied: time for mercury deposition on the gold electrode surface, imposed current for mercury dissolution and electrode surface area.

A calibration straight line was drawn for mercury determination by stripping chronopotentiometry within 5 – 100 μg/L range (y = 0.163x – 0.624, R^2^ = 0.994) for a deposition time of 180 s. RSD was 1.92% (n=6) for mercury concentration of 20 μg/mL. Mercury could be measured also at lower concentrations. A calibration straight line was drawn within 1 – 10 μg/L range (y = 281x + 0.226, R^2^ = 0.988) for a deposition time of 600 s. Detection limit was 0.30 μg/L and determination limit was 1.0 μg/L for a deposition time of 600 s.

A method for fish sample digestion was optimized by using a mixture of fumans nitric acid and, both concentrated, sulfuric and hydrochloric acids. The sample were analysed after digestion by stripping chronopotentiometry by means of the developed working electrode. A recovery test was applied in order to check the results against an standard amount of mercury introduced in the sample before digestion and the standard addition method was applied. The calculated recovery degree was 95.3% (n=4). The results obtained by the proposed method were compared with those obtained by atomic absorption spectrometry. The concordance for mercury determination by using the two methods was good. Gold electrodes made from CD-R's are much cheaper than commercially available gold electrodes. Such an electrode can be used for approximately 40 experimental runs.

## Figures and Tables

**Table 1.** Results of mercury determination for a cod sample analysis by the proposed method and CVAAS.

**Sample amount (g)**	**Proposed method(μg/g)^a^**	**Recovery(%)^b^**	**CVAAS(μg/g)^a^**	**Recovery(%)^b^**
Cod (2.0083 g)	0.239±0.012	-	0.228±0.008	-
Cod (2.0076 g +0.6 μg Hg (standard solution))	0.524±0.021	95.3	0.520±0.008	97.6

a) All values represent a mean for four measurements ± *k* x standard deviation (where *k*=3).

b) Recovery. Mercury recovery was calculated by determining the mercury amount (μg/g) in a cod sample and in another one to which a known amount of mercury was added (μg/g). The difference between these two quantities represents the recovered mercury. Ratio between recovered mercury to the added one gives the recovery degree.
